# Retrospective Study of Vitreous Tap Technique Using Needle Aspiration for Management of Shallow Anterior Chamber during Phacoemulsification

**DOI:** 10.1155/2017/2801025

**Published:** 2017-05-09

**Authors:** Ashraf Ahmed Nossair, Wael Ahmed Ewais, Lamia Samy Ali

**Affiliations:** ^1^Department of Ophthalmology, Faculty of Medicine, Cairo University, Kasr Al Ainy Street, Cairo 11562, Egypt; ^2^Dar El Oyoun Hospital, 9 Ghazali Street, Dokki, Giza 12611, Egypt

## Abstract

**Purpose:**

To evaluate the technique of vitreous tap using needle aspiration for management of anterior chamber shallowness during phacoemulsification.

**Methods:**

A retrospective study included 26 eyes of 17 patients who underwent phacoemulsification in which vitreous tap was performed using a 27-gauge needle attached to a 5 ml syringe, inserted 3.5 mm from the limbus to aspirate 0.2 ml of liquefied vitreous if a cohesive (OVD) failed to sufficiently deepen the anterior chamber.

**Results:**

Preoperative anterior chamber depth was 2.31 ± 0.26 mm, axial length was 21.7 ± 0.67 mm, lens thickness was 4.5 ± .19 mm, and cataract grade was 3.77 ± 1.4. Preoperative CDVA in LogMAR units was 0.98 ± 0.75. Coexisting angle closure glaucoma was present in 7 patients (26.92%) preoperatively. Vitreous needle tap was successful in vitreous removal on the first attempt in 26 eyes (100%). Postoperative follow-up period was 22.88 ± 10.24 (4–39) months. The final postoperative CDVA in LogMAR units was 0.07 ± 0.1, while the final postoperative IOP was 16.54 ± 1.45 mmHg. No complications related to vitreous tap were noted.

**Conclusion:**

Vitreous needle tap is a simple, cost-effective, and safe technique for management of shallow anterior chamber in phacoemulsification.

## 1. Introduction

During phacoemulsification, shallow anterior chamber interferes with almost every step, starting from the creation of wound incisions and capsulorhexis till the intraocular lens implantation [1].

Working in a narrow space inside the eye increases the risks of Descemet's membrane detachment, capsulorhexis extension, and zonular dialysis. High-vitreous pressure in such eyes can result in iris prolapse or posterior capsular rupture with subsequent vitreous loss and possibly suprachoroidal haemorrhage. Furthermore, corneal endothelial cell loss is a main concern due to the closer distance between phacoemulsification tip and corneal endothelium [1–5]. Postoperative intraocular pressure elevation, macular oedema, choroidal effusion, and malignant glaucoma develop more frequently in these eyes [1, 4–6].

Shallow anterior chamber is commonly seen in eyes with short-axial length. The term small eye is used to describe a spectrum of disorders characterized by short-axial length [6]. At the extreme end of the spectrum, there are nanophthalmos and complex microphthalmos which are rare diseases characterized by multiple ocular malformations [6–9]. Relative anterior microphthalmos is more common and characterized by normal axial length, small corneal diameter, and anterior segment [6, 10].

Shallow anterior chamber with normal axial length can be seen in the presence of angle closure glaucoma with or without peripheral anterior synechiae [1, 4], intumescent cataract [3, 11], and subluxated lens [12, 13].

Ocular or systemic hypertension, arteriosclerosis, chronic obstructive pulmonary disease, senility, and obesity may precipitate positive vitreous pressure and anterior chamber shallowness during cataract surgery [1].

Small eyes with shallow anterior chamber are commonly associated with other problems such as narrow palpebral fissure, small pupil, large lens, and narrow angle glaucoma [4–6]. Cases of intumescent cataract may present with phacomorphic glaucoma and absent red reflex [3, 11]. The aforementioned complexity creates additional difficulty to these cases.

Preoperative use of dehydrating agents decreases intraocular pressure and deepens the anterior chamber [4, 6]. Intraoperative uses of a cohesive ophthalmic viscoelastic device (OVD) [14] or an anterior chamber maintainer [1] are also helpful methods.

However, performing limited pars plana vitrectomy to remove small volume of vitreous is considered the only possible way to successfully deepen the anterior chamber in some cases [1–3, 13]. Although vitrectomy is a valuable option, it is not free of drawbacks.

On the other hand, diagnostic vitreous biopsy using needle aspiration was proved to be safe and effective in cases of endophthalmitis [15], intraocular inflammations [16], and malignancy [17].

In this study, we report on the use of vitreous needle aspiration in cases of shallow anterior chamber during phacoemulsification to evaluate its safety and efficacy.

## 2. Patients and Methods

### 2.1. Study Design

A retrospective uncontrolled study included 26 eyes of 17 patients who underwent vitreous tap using needle aspiration to manage shallow anterior chamber during phacoemulsification, after reviewing the records of 612 eyes operated upon between May 2013 and May 2016 in a tertiary care hospital by one surgeon (A. Nossair) who had been adopting the use of this technique for several years. Informed consent for surgery was obtained from all patients. Study protocol followed the Declaration of Helsinki and was approved by an institutional review board.

### 2.2. Standard Surgical Technique

Pupil dilation was done using the standard topical tropicamide 1% and phenylephrine 2.5% instilled every 10 minutes, one hour before surgery.

Peribulbar local anaesthetic mixture of lidocaine 1% and bupivacaine 0.5% was injected, followed by 20 minutes of external ocular massage. Temporal corneal incision of 2.8 mm width and two side ports were created. The anterior chamber was filled with a cohesive ophthalmic viscoelastic device (OVD), Healon5® (Abbott Medical Optics Inc., CA, USA). Four iris hooks were used in cases of poorly dilated pupil (defined as less than 6 mm diameter). Continuous curvilinear capsulorhexis of 5 to 5.5 mm diameter was completed. Trypan blue capsular staining was used in intumescent cataract cases. Phacoemulsification was done by phaco chop technique using Infiniti phacoemulsification machine (Alcon Labs, Fort Worth, TX, USA). Irrigation aspiration of remaining cortical material and foldable intraocular lens implantation in the capsular bag was achieved in all cases.

Intraoperative complications were recorded. Ultrasound energy and total phacoemulsification time were also recorded to calculate effective phacoemulsification time (EPT).

### 2.3. Vitreous Tap Using Needle Aspiration Technique

Vitreous aspiration was done using a 27-gauge needle attached to a 5 ml syringe, inserted through pars plana, 3.5 mm from the limbus, directed towards the optic nerve to slowly aspirate an amount of 0.2 ml. Vitreous needle aspiration was indicated if cohesive ophthalmic viscoelastic device failed to expand the anterior chamber initially after corneal incision and before capsulorhexis. If the first attempt of aspiration failed to aspirate vitreous fluid or was insufficient to adequately deepen the anterior chamber in spite of successful vitreous removal, a second vitreous tap was done at a different site. Failure of the technique was defined as failure of two attempts to deepen the anterior chamber. The technique was not done in patients who were younger than 40 years old as they had formed vitreous or in the presence of posterior segment pathology such as vitreous haemorrhage or retinal detachment.

### 2.4. Retrospective Chart Review

Preoperative and demographic data were retrieved. Cataract grading had been assessed using Lens Opacities Cataract Classification System III (LOCS III) [18]. Preoperative anterior chamber depth, lens thickness, and axial length had been measured by ultrasonic device, Sonomed E-Z AB5500 (Sonomed Inc., USA). Hoffer Q formula had been used to calculate intraocular lens power. Recorded preoperative and postoperative Snellen corrected distance visual acuity (CDVA) was converted to logarithmic minimal angle of resolution (LogMAR) units for statistical analysis. Intraoperative data such as effective phacoemulsification time (EPT), site of vitreous tap, number of vitreous tap attempts, aspirated vitreous volume, and intraoperative complications, if any, were collected. Preoperative and postoperative spherical equivalent, intraocular pressure, and complications were also reviewed.

### 2.5. Statistical Analysis

All data were pooled into a central database. Statistical analysis was performed using SPSS software package (version 17.0, SPSS Inc., Chicago, IL, USA). Means ± standard deviations (SD) and ranges were used for descriptive data. The Pearson coefficient was used for the correlation between different variables. *P* value <0.05 was considered significant.

## 3. Results

### 3.1. Base Line Characteristics

Twenty-six eyes of 17 patients were included in this study. The right eye was involved in 14 eyes (53.85%), and the left eye was involved in 12 eyes (46.15%). The females represented 17 eyes (65.38%), and the males represented 9 eyes (34.62%). The average age was 60.8 ± 8.19 (47–79) years.

Preoperative anterior chamber depth was 2.31 ± 0.26 (range 1.73 to 2.72) mm, axial length was 21.7 ± 0.67 (range 20.43 to 22.64) mm, and lens thickness was 4.5 ± .19 (range 4.22 to 4.87) mm. Cataract grade was 3.77 ± 1.4 (range 2 to 6) using Lens Opacities Cataract Classification System (LOCS) III.

Eight eyes (30.77%) were not refractable preoperatively due to dense cataract (intumescent cataract). Spherical equivalent of the remaining 18 eyes (69.23%) was +5.9 ± 2 (range + 3.25 to +9.5) diopters (D). Preoperative CDVA in LogMAR units was 0.98 ± 0.75 (0.3–3).

The coexisting primary angle closure glaucoma was present in 4 eyes (15.38%), and the secondary angle closure glaucoma (phacomorphic glaucoma) was present in 3 eyes (11.54%), with a total of 7 glaucomatous eyes (26.92%) preoperatively.

### 3.2. Intraoperative Results

The small pupil was noted in 10 eyes (38.46%). The iris hooks were used intraoperatively to dilate the pupil in theses eyes.

Vitreous needle tap was successful in vitreous removal on the first attempt in 26 eyes (100%). Despite of successful vitreous removal in all eyes, anterior chamber depth was still considered shallow in 2 eyes. An additional vitreous tap was required in these 2 eyes to get an adequate depth with a total of 28 successful vitreous tap attempts. A zero failure rate of the technique was observed. The site of vitreous tap was inferotemporal quadrant in 14 times (50%) and supertemporal quadrant in 14 times (50%).

**Figure 1:**
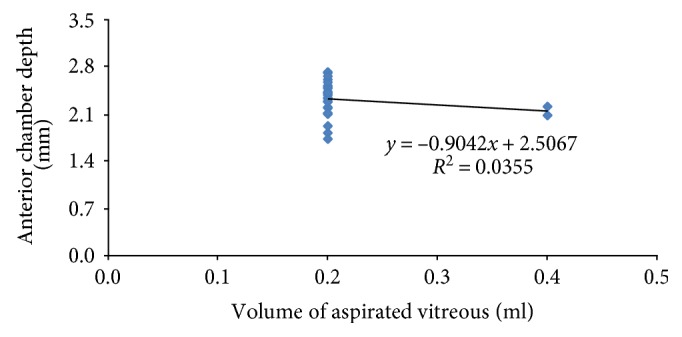
Correlation between preoperative anterior chamber depth and aspirated vitreous volume.

The average volume of aspirated vitreous was 0.215 ± 0.05 (0.2–0.4) ml. There was a negative correlation between preoperative anterior chamber depth and aspirated vitreous volume which was statistically insignificant (*R* = 0.19, *P* = 0.36), (Figure 1).

Effective phacoemulsification time (EPT) was 6.62 ± 1.44 (range 4.5 to 9.5) seconds.

**Figure 2:**
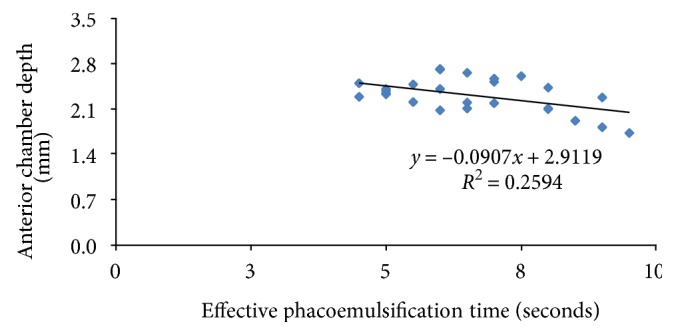
Correlation between anterior chamber depth and effective phacoemulsification time.

There was a strong negative correlation between preoperative anterior chamber depth and effective phacoemulsification time. This was statistically significant (*R* = 0.5, *P* = 0.0079), (Figure 2). No intraoperative complication was observed whether related to vitreous tap or the standard phacoemulsification procedure.

### 3.3. Postoperative Results

The postoperative follow-up period was 22.88 ± 10.24 (4–39) months. The final postoperative CDVA in LogMAR units was 0.07 ± 0.1 with a range between −0.1 and 0.2, while the final postoperative spherical equivalent was +0.27 ± 0.87 with a range between −1.0 and +1.75 D. The postoperative intraocular pressure (IOP) was 16.54 ± 1.45 (range 14 to 19) mmHg. Two glaucomatous patients needed timolol 0.5% eye drops to control intraocular pressure postoperatively. Mild striate keratopathy (SK) was observed in two patients in the early postoperative period and resolved completely within two weeks.

## 4. Discussion

Extreme caution is recommended when operating on eyes with shallow anterior chamber. Proper preoperative evaluation allows better planning of the surgery as preventive measures should be considered to avoid complications in these cases.

Perioperative measures may include infusion of intravenous mannitol 30 minutes to one hour before surgery to decrease positive vitreous pressure [4, 6], but its use is largely limited by its serious systemic side effects.

In this study, peribulbar anaesthesia was used for all patients. It offered the benefits of abolishing the action of extraocular muscles and giving enough duration of anaesthesia if the procedure was prolonged. Furthermore, the injected fluid may lift the globe upwards, improving surgical exposure in cases of deeply set eyes. The downside of peribulbar anaesthesia is that it may increase orbital pressure, but injecting minimal anaesthetic fluid volume and orbital decompression by external ocular massage for 20 minutes should counteract this problem [19]. Some surgeons prefer topical anaesthesia or general anaesthesia in these cases as both methods do not increase vitreous or orbital pressure. However, topical anaesthesia does not provide extraocular muscle akinesia and depends on patient cooperation. While general anaesthesia can provide extraocular muscle paralysis without any increase in orbital pressure, it is not suitable for all patients and it carries the potential cardiovascular and respiratory risks of general anaesthesia [5, 6].

Slightly elevated head position may decrease venous and vitreous pressures. It is also important to make sure that the eye lid speculum is not exerting any pressure on the globe [19]. Fashioning of well-beveled corneal incisions is crucial to maintain anterior chamber space by preventing wound leakage. Temporal incisions in this study were created to increase surgical exposure and to deal better with the commonly encountered narrow palpebral fissure and deeply set eyes in these patients.

Cohesive ophthalmic viscoelastic device (OVD) use is highly advised [6, 14]. Soft-shell technique by adding a dispersive (OVD) to a cohesive (OVD) has been described to add more corneal protection [20]. In this study, Healon5 was used as it has both cohesive and dispersive properties. It is considered as a viscoadaptive formula owing to its high viscosity and elasticity in presence of low- and high-shear rates. When exposed to phaco power and continuous fluid turbulence, it acts like a dispersive OVD by fragmentation and formation of a cavity while retaining an outer protective shell. Thus, it can maintain anterior chamber depth and protect the cornea at all surgical steps [21]. However, despite considering all of these measures in our study, an anterior vitreous tapping was necessary to get a satisfactory depth of anterior chamber.

A relatively large capsulorhexis was done to perform phacoemulsification without stressing zonular attachments, followed by minimal hydrodissection to avoid inadvertent vitreous hydration. Elevation of bottle height and decreasing flow rate were also considered to stabilize anterior chamber depth. Phaco chopping technique was used in the study as it utilizes less ultrasonic energy to decrease corneal endothelial damage.

Management of the commonly associated difficulties was mandatory for successful results. The small pupils were dilated using iris hooks instead of pupillary rings or expanders which are more hazardous to the cornea [6], and capsular staining was performed by Trypan blue in white cataracts.

Chang in 2001 described a single-port-limited pars plana vitrectomy without infusion cannula using a 20-gauge vitrectomy probe inserted through a sclerotomy incision 3.5 mm from the limbus for management of crowded eyes in phacoemulsification [2]. Small gauge vitrectomy was used later for these eyes [1, 3, 13], (Table 1).

Although vitrectomy is a valuable option, it has some disadvantages. The fashioned sclerotomy may leak or require suturing. That is why using the small 23 or 25 gauge vitrectomy probe is preferred to using the conventional a 20-gauge vitrectomy probe for this purpose, in addition to the advantage of higher cutting rates resulting in minimal retinal traction, but unfortunately most of the phacoemulsification systems incorporate low-cutting speed 20-gauge vitreous cutters unless combined anterior and posterior segment phacoemulsification and vitrectomy systems are used, which add significantly to the cost of surgery due to their expensive disposable consumables. This could be an important issue in case of limited resources such as in developing countries. Furthermore, removal of too much vitreous by this technique may produce too deep anterior chamber which is undesirable.

Several authors have investigated vitreous tap using needle aspiration in diagnosis of endophthalmitis [15], uveitis [16], and intraocular tumours [17] and suggested its safety. The main fear of vitreous needle aspiration is inducing retinal traction with subsequent retinal tears, vitreous haemorrhage, or retinal detachment [19]. In the large multicentre endophthalmitis vitrectomy study, the results showed that there was no significant difference between vitreous needle aspiration and automated vitrectomy regarding posterior segment complications and the final visual outcome over a follow-up period of 9 to 12 months [15], (Table 2).

The idea of using vitreous needle aspiration to manage positive vitreous pressure during surgery was investigated previously in penetrating keratoplasty (PKP) [22] and in triple procedure involving PKP, cataract extraction, and intraocular lens implantation [19]. The technique was found to be safe without any complications.

Infusion misdirection syndrome describes acute intraoperative anterior chamber shallowness and intraocular pressure rise in absence of choroidal effusions. It occurs due to migration of irrigating fluid via zonular fibres to reach the posterior segment [23]. This usually occurs after excessive hydrodissection or during irrigation aspiration of cortical remnants. It was also described as acute aqueous misdirection syndrome to differentiate it from malignant glaucoma (aqueous misdirection syndrome) which has similar clinical picture but occurs later in the postoperative period [24]. Lau et al. named this condition acute intraoperative rock-hard eye syndrome (AIRES) and managed it successfully by vitreous needle aspiration [23]. It is of utmost importance to exclude other causes of intraoperative shallow anterior chamber such as choroidal effusion or suprachoroidal haemorrhage before doing vitreous needle aspiration or vitrectomy in such cases [14], (Table 3).

Vitreous tap for management of crowded eyes in cataract surgery was suggested earlier using a 23- to 26-gauge needle attached to an insulin syringe without the plunger to allow passive removal of vitreous. This technique avoids vitreous aspiration which may induce traction on the retina [25]. However, passive vitreous flow will not occur unless a liquefied vitreous lacuna was located by the needle, otherwise a searching movement may be required which can be hazardous. The previous technique was described by the authors in a letter to the editor without studied patient results [25]. In this study, vitreous tap using vitreous needle aspiration was performed to remove a 0.2 ml of vitreous, which was believed to be more successful in vitreous removal in most of cases without increasing the risks of retinal traction as the aspirated volume was minimal. Vitreous tap using needle aspiration is machine independent. It uses simple needles and syringes that are readily available in any operating room. In addition of being easy to perform, it reduces costs and saves time without creating an extra wound while allowing a precise amount of vitreous to be removed. Our results showed that it is a reproducible technique as it was successful in removing the vitreous on the first attempt in all patients. The technique could be repeated during surgery without drawbacks. No failure rate was detected.

In this study, no major intraoperative or postoperative complication was observed over a relatively long postoperative follow-up period. This is contradictory to the higher incidence of posterior segment complications in studies involving diagnostic vitreous needle aspiration which can be explained by the pathological vitreous condition in diseases like endophthalmitis. This is further evidenced by the similar absence of posterior segment complications related to retinal traction in the previously mentioned studies for management of positive pressure in acute intraoperative fluid misdirection syndrome, PKP, and triple procedures. Another important factor is the presence of physiological age-related vitreous liquefaction in our study group of patients.

Vitreous needle aspiration can safely manage cases of shallow anterior chamber due to short-axial length, angle closure glaucoma, intumescent cataract, or acute intraoperative fluid misdirection. Using this technique can preserve corneal endothelial cells and posterior capsular integrity. Another benefit is preventing postoperative malignant glaucoma which may occur in these eyes. This technique is better avoided in young patients or in cases of coexisting posterior segment pathology.

It is worth mentioning that recent trends in modern ophthalmology are expanding the indications of lens extraction surgery such as refractive lens exchange for hyperopia [26] and clear lens extraction for angle closure glaucoma [27–29]. Thus, it is anticipated that phacoemulsification surgeons will face an increasing number of cases with shallow anterior chamber in the near future.

## 5. Conclusion

This study suggests that vitreous tap using needle aspiration is a simple, cost-effective, rapid, and safe technique for management of shallow anterior chamber in phacoemulsification. Further studies may confirm these results.

## Figures and Tables

**Table 1:** Studies suggesting using limited pars plana vitrectomy for management of anterior chamber shallowness in phacoemulsification.

	Indications	Number of patients (*n*)	Vitrectomy gauge
Chang [2]	Chronic angle closure glaucoma	4	20 gauge
Chalam et al. [1]	Positive vitreous pressure	Not specified	25 gauge
Dada et al. [3]	Phacomorphic glaucoma	2	25 gauge
Miura et al. [13]	Acute angle closure glaucoma	17	25 gauge

**Table 2:** The results of vitreous needle tap versus automated vitreous biopsy in the endophthalmitis vitrectomy study according to Han et al. [15].

	Gauge size	Number of patients (*n*)	Intraoperative hyphema	Postoperative retinal detachment	Culture and gram stain positivity
Vitreous needle tap	20	70	2 (3%)	8 (11%)	69% and 42%
Automated vitreous biopsy	22–27	127	3 (2%)	10 (8%)	66% and 41%

**Table 3:** Studies suggesting using vitreous needle aspiration to manage positive vitreous pressure during intraocular surgery.

	Intraocular procedure	Number of patients (*n*)	Needle gauge and syringe used
Gross & Shaw [22]	Penetrating keratoplasty (PKP)	70	21-gauge needle on a 3 ml syringe
Vongthongsri et al. [19]	Triple procedure (PKP, cataract extraction, and lens implantation)	65	23-gauge needle on a 5 ml syringe
Lau et al. [23]	Phacoemulsification	6	23-gauge on a 3 ml syringe
